# Genetic susceptibility to total hip arthroplasty failure: a case–control study on the influence of MMP 1 gene polymorphism

**DOI:** 10.1186/s13000-014-0177-9

**Published:** 2014-09-26

**Authors:** Yiguo Yan, Jianzhong Hu, Hongbin Lu, Wenjun Wang

**Affiliations:** Department of Spine Surgery, Xiangya Hospital, Central South University, No:87 Xiangya Road, Changsha, Hunan People’s Republic of China; Department of Spine Surgery, The First Affiliated Hospital of University of South China, Hengyang, People’s Republic of China

**Keywords:** Aseptic loosening, SNP, MMP-1

## Abstract

**Background:**

Genetic factors plays an important role in early failure of total hip arthroplasty (THA) etiology and MMP-1 gene polymorphism rs5854 may be involved. The present study was conducted to reveal the possible association between MMP-1 rs5854 C/T polymorphism and the risk of early failure of THA (aseptic loosening).

**Methods:**

The rs5854 single nucleotide polymorphism (SNP) in MMP-1 gene was genotyped in 63 subjects who were diagnosed as aseptic loosening after total hip arthroplasty within 10 years and in 81 age and gender matched controls.

**Results:**

The genotype frequencies of the MMP-1 rs5854 C/T polymorphism were 57.1% (CC), 28.6% (CT), and 14.3% (TT) in patients with failure of THA, and 79.0% (CC), 17.3% (CT), and 3.7% (TT) in the controls (P = 0.0099). Rs5854 polymorphism was found to be significantly associated with increased risk of aseptic loosening.

**Conclusion:**

The results showed the rs5854 SNP was associated with increased risk of the early aseptic loosening susceptibility.

**Virtual Slides:**

The virtual slide(s) for this article can be found here: http://www.diagnosticpathology.diagnomx.eu/vs/13000_2014_177

## Background

Total hip arthroplasty (THA) has become the gold standard treatment for patients with end stage arthritis [1]. Multiple studies have demonstrated that THA improved the physical function and quality of life significantly [2-4]. Indications for THA have increased in the last decades, along with the number of procedures performed annually. Almost 1 million of THA was implanted worldwide annually, with a predicting increase of 174% to nearly 600,000 THA procedures annually by 2030 in the United States [5-7]. However, indispensable proportion of patients after THA still face the complications that may lead to the premature prosthesis failure and revision surgery, with a significant impact on quality of life [5]. While sepsis, fracture, and dislocation are relatively rare, aseptic loosening that arising from aseptic inflammatory reactions to the prosthetic implants accounts for 75.7% of all THA revisions [8-10]. The implanted prosthesis stimulates mesenchymal cells to inflammatory response and osteoclast accumulation, leading eventually to excessive resorption, bone loss, and periprosthetic osteolysis [11,12].

The loosening of prosthetic implants can be attributed to biological, physical and biophysical factors, however, the precise aetiology remains unclear. At present, it is thought that the individual difference of susceptibility to aseptic loosening results from a combination of environmental and genetic factors. Environmental factors include type of prosthesis, implant design, material, surgical technique, fixation method, and postoperative rehabilitation procedure have been widely studied [13,14], but more and more attention is paid to the genetic factors like single nucleotide polymorphisms (SNPs). SNPs are genetic variations that are considered biologically normal and can be found in at least 1% of the population, which may contribute towards individual susceptibilities to certain pathological conditions [15,16]. Various gene SNPs of GNAS1, TNF-238, TNF-a, IL6-174, MMP1, MMP2, and et al. were reported to be associated with increased prosthetic loosening.

Matrix metalloproteases (MMPs), which are secreted by inflammatory cells in response to stimuli from lipopolysaccharides and cytokines [17], are the biggest class of enzymes responsible for metabolism of the extracellular matrix(ECM) [18]. MMP-1 performs an important function in collagen degradation including collagen types I, II, and IX, which are the most abundant protein components of the ECM [18-21]. Previous researches have demonstrated that proteases were present in the peri-implant fluid and might perform a pathological role in peri-implant bone loss [21]. Interstitial collagenase follows the osteoblasts at the start of the bone reabsorption, thus generating collagen fragments and activating the osteoclasts [22]. MMP-1 is usually expressed at low levels but it is induced by phorbol esters, growth factors, and inflammatory cytokines [23]. Previous studies have demonstrated the polymorphism in the promoter of the MMP-1 gene was associated with early implant failure of THA [24]. Similarly, the C allele and C/C genotype of the MMP-1 SNP rs5854 was found highly associated with aseptic failure [25]. However, further research is needed to replicate previous findings. This study aims to determine whether the MMP-1 SNP rs5854 was associated with failure of THA (aseptic loosening) in Chinese Han Populations.

## Method

This study was approved by the ethics committee of the Xiangya Hospital, and informed consent was obtained from all the patients and control participants.

### Study population

The present study enrolled 63 patients who were diagnosed as aseptic loosening of prosthetic hip joints at the Department of Orthopaedics of Xiangya Hospital, China. Strict inclusion and exclusion criteria was used for all these patients. Entry criteria for this study including clinical, radiological, laboratory, and intrasurgical diagnosis of aseptic loosening within the first 10 years after total hip arthroplasties. The diagnostic criteria for aseptic loosening of the prosthesis was listed as the following: 1. Hip pain when walking or moving the joint. 2. Migration of prosthetic components or bone radiolucency around the prosthesis of more than 2 mm. 3. Inflammatory tests within normal patterns — erythrocyte sedimentation rate, polymerase chain reaction (PCR), and leukogram. Patients were excluded if they had any deep infection or the suspicion of implant infection, traumatic loosening, inflammatory diseases, or immunosuppress ant agents after THA in their history. The control group consists of 81 age- and gender- matched patients who had undergone THA that had been proved to be therapeutically successful over long-term follow-up (at least 10 years). All subjects included in this study were Chinese Han Population.

### Genotyping

DNA samples were obtained from all the participants from peripheral blood with the Chelex-100 method [26]. The MMP-1 SNP rs5854 was then genotyped using Taqman assay (Applied Biosystems 7500, ABI, Foster City, CA) and dual-labeled probes in real-time PCR. The primers and probes were designed and synthesized by Sigma (Sigma-Proligo, The Woodlands, TX). Genotyping was performed by independent laboratory personnel who was blinded to the study, and three authors independently reviewed the genotyping results, data entry, and statistical analyses. In addition, we randomly selected 5% samples of case and control subjects for reproducibility tests at least twice in different days and yielded a 100% concordant.

### Statistical analysis

The Statistical Package for Social Sciences software (SPSS, Inc., Chicago, IL, USA), version 16.0 for Windows. The demographic and clinical data were presented as Mean ± SD and compared between groups by the Student’s t‑tests and Chi‑square test. The genotype and allelic frequencies were evaluated by Hardy‑Weinberg equilibrium and compared by the Chi‑square test. The association between the MMP-1 rs5854 polymorphism and failure of THA was assessed under the following genetic models, which were treated as a dichotomous variable: (i) T-allele versus C-allele for allele level comparison; (ii) CT + TT versus CC for a dominant model of the T allele; (iii) TT versus CT + CC for a recessive model of the T-allele; and (iv) TT versus CC for the extreme genotype. Multivariate logistic regression was used to estimate odds ratios (ORs) and 95% confidence intervals (CI) after adjustment for age and gender. The P < 0.05 was considered to indicate a statistically significant difference.

## Results

### Patient characteristics

Demographic data of the population studied and the number of individuals in each group were shown in Table 1. There were no significant differences between groups in terms of age, gender, and BMI. There were 17 patients with a early aseptic loosening of the total hip prosthesis during the first five years after surgery. And the remaining 46 patients developed aseptic failure during five to ten years after surgery.

**Table 1:** **The summary of the basic characteristics of the groups**

**Clinical characteristics**	**Aseptic loosening patients**	**Controls**	**P-value**
No.	63	81	
Female/Male	28/35	33/48	n.s
Age (years, Female/Male)	70.8 ± 9.7/71.6 ± 9.0	72.0 ± 6.3/72.6 ± 7.1	n.s
BMI (kg/m^2^)	28.2 ± 6.2	27.7 ± 6.9	n.s
Period before aseptic loosening (years)		/	/
0-5	17	/	/
5-10	46	/	/

### Association of MMP-1 rs5854 polymorphism with susceptibility to failure of THA

As expected, the distribution of the genotype of MMP-1 SNP rs5854 conformed to the Hardy–Weinberg equilibrium and the genotyping success rate was 100% Table 2 listed the genotyped and allele distributions of the SNP rs5854 for the cases and controls. The genotype frequencies of the MMP-1 rs5854 C/T polymorphism were 57.1% (CC), 28.6% (CT) and 14.3% (TT) in failure of THA patients, and 79.0% (CC), 17.3% (CT) and 3.7% (TT) in controls (P = 0.0099). Multivariate logistic regression was used to estimate odds ratios (ORs) and 95% confidence intervals (CI) after adjustment for age and gender. For allele level comparison, the MMP-1 SNP rs5854 T allele was associated with an increased risk of aseptic loosening in terms of the frequency of allele comparison (T vs. C:OR = 2.72; 95% CI =2.06 – 3.48, P <0.0001). For a dominant model of the T allele, the CT + TT genotypes were associated with the risk for aseptic loosening (CT + TT vs. CC, OR = 2.67, 95% CI = 1.25 - 5.76, P = 0.0028). For a recessive model of the T allele, the TT homozygote genotype was associated with susceptibility to aseptic loosening (TT vs. CT + CC. OR = 4.17, 95% CI =1.22 - 17.2, P = 0.0186). For the extreme genotype, the TT genotypes were associated with the risk for aseptic loosening (TT vs. CC, OR = 5.13, 95% CI = 1.33 to 19.8 P = 0.0113).

**Table 2:** **The genotype and allele distributions of the MMP-1 SNP rs5854 for the cases and controls**

**Group**	**Allele (%)**								
	**CC**	**CT**	**TT**	**CT + TT**	**CT + CC**	**TT**	**C**	**T**	**H-WE**
Control	64	14	3	17	78	3	87.7	12.3	n.s
Case	36	18	9	27	54	9	71.4	28.6	/
OR^a^ (95% CI)	/	/	5.13 (1.33 to 19.8)	2.67 (1.25 to 5.76)	/	4.17 (1.22 to 17.2)	/	2.72 (2.06 to 3.48)	/
P^a^	/	/	0.0113	0.0028	/	0.0186	/	< 0.0001	/

^a^ORs and 95% CIs were estimated using multiple logistic regression analyses and adjusted for age, gender and BMI.

Additionally, the association analysis was also performed in regarding with the period before aseptic loosening happens. Significant association was found in terms of the comparison of the frequency of allele and comparison with a dominant model. However, no significant association were found with the dominant model and for the extreme genotype comparison Table 3.

**Table 3:** **Subgroup analysis of the MMP-1 SNP rs5854 regarding with the period before aseptic loosening happens**

**Period (year)**	**Allele (%)**							
	**CC**	**CT**	**TT**	**CT + TT**	**CT + CC**	**TT**	**C**	**T**
0-5	5	8	4	12	13	4	52.9	47.1
5-10	31	10	5	15	41	5	78.3	21.7
OR^a^ (95% CI)	/	/	0.23 (0.12 to 1.16)	0.15 (0.08 to 0.43)	/	0.37 (0.09 to 1.47)	/	0.28 (0.10 to 0.40)
P^a^	/	/	0.0750	0.0025	/	0.1762	/	< 0.0001

^a^ORs and 95% CIs were estimated using multiple logistic regression analyses and adjusted for age, gender and BMI.

## Discussion

The present study was conducted to reveal the possible association between MMP-1 rs5854 C/T polymorphism and the risk of early failure of THA (aseptic loosening). The results showed the rs5854 SNP was associated with increased risk of the aseptic loosening susceptibility.

Although total hip prosthetic implants improve quality of life significantly for most patients, they are not built to last forever. Importantly, aseptic loosening due to the periprosthetic osteolysis is known to be the main complication of early failure after THA. Aseptic loosening has been considered to introduced by biological, microbiological, and biomechanical factors. However, the mechanisms and causes of early failure of THA results from aseptic loosening continue to be a matter of study. Nowadays, the abnormal immunological responses were considered as the core of the pathologies.

Abnormal immunological response that participated in destroy of the peri-implant tissues involves almost all immunological cells like macrophages, endothelial cells, fibroblasts, osteoclasts, and osteoblasts [27,28]. When activated, these cells may synthesize various cytokines and lipid mediators that involve inflammatory and aseptic periprosthetic osteolysis processes [13,29,30]. Though the inflammatory cytokines trigger a chronic inflammatory status, it may also determine soft tissue and supporting bone damage if untreated [8,9].

MMPs is one of the cytokines and lipid mediators that increase the destruction of the peri-implant proteolytic tissue [31-33]. The effect of MMPs may be produced by the direct degradation of the extracellular organic matrix of bone. The expression of MMP-13 was found in macrophages, endothelial cells, and fibroblasts of the synovial-like membrane around the implant [34]. Local mRNA expression profile also demonstrated that MMP-1, −9, −10, −12 and −13 were strongly elevated in aseptic loosening compared to the controls; MMP-2, −7, −8, −11, −14, −15, −16, −17 and −19 were moderately expressed, whereas MMP-3 expression was lower and MMP-20 very low [35]. Furthermore, collagen degradation in periprosthetic tissue correlated significantly with the number of local MMP-1, MMP-13 and cathepsin K-positive cells [36].

Individual susceptibility to aseptic loosening after THA is partly determined by patient-related factors, namely, the genetic factors. There is evidence that the individual differences may be contributed to the polymorphisms of various genes encoding cytokines involved in the development of aseptic loosening [24,37,38]. Polymorphism of the genes that express MMPs is associated with development of ovarian cancer, endometrial carcinoma, changes in tooth mineralization, premature rupture of the fetal membrane and severe periodontiti [19,20,39,40]. Recently, Malik and co-workers reported the association of a MMP-1 SNP rs5854 and the occurrence of increased aseptic loosening susceptibility [25]. Similarly, this study demonstrated positive association between MMP-1 SNP rs5854 and aseptic loosening susceptibility, moreover, it seemed also to be associated with earlier occurrence of aseptic loosening.

The MMP-1 SNP rs5854 exists within a promoter region of the gene and thus have a direct effect on the amount and function of gene expression [41]. However, the exact mechanisms and effects of SNP rs5854 on MMP gene regulation are still not fully delineated. Proteolysis of extracellular matrix components can affect cell–cell communication and cell apoptosis. Degradation products of collagen and matrix components are chemotactic for monocytes, which may lead to the aggravation of inflammatory reaction in the periprosthetic tissues.

The present study demonstrated MMP-1 rs5854 C/T polymorphism was associated with increased risk of early failure of THA (aseptic loosening). It may be help in understanding the pathologies of aseptic loosening. Moreover, the evaluation of MMP-1 rs5854 SNP may be help in the diagnosis and prevention of aseptic loosening. However, there is some limitation in the present study. The most important is the relatively small sample size. The subgroup analysis according to the period before aseptic loosening happens did not show significant association, which may be result from the relative small sample size in the groups. Additionally, a single center case– control study is not sufficient to fully interpret the relationship between MMP-1 polymorphisms and susceptibility to aseptic loosening. Further prospective study with multiple population and larger sample size is needed.

## Conclusion

The present study was conducted to reveal the possible association between MMP-1 rs5854 C/T polymorphism and the risk of early failure of THA (aseptic loosening). The results showed the rs5854 SNP was associated with increased risk of the early aseptic loosening susceptibility.
